# Validating Excised Rodent Lungs for Functional Hyperpolarized Xenon-129 MRI

**DOI:** 10.1371/journal.pone.0073468

**Published:** 2013-08-30

**Authors:** David M. L. Lilburn, Theodore Hughes-Riley, Joseph S. Six, Karl F. Stupic, Dominick E. Shaw, Galina E. Pavlovskaya, Thomas Meersmann

**Affiliations:** 1 Sir Peter Mansfield Magnetic Resonance Centre, School of Clinical Sciences, University of Nottingham, Nottingham, United Kingdom; 2 Nottingham Respiratory Research Unit, Nottingham City Hospital, Nottingham, United Kingdom; Bascom Palmer Eye Institute, University of Miami School of Medicine; United States of America

## Abstract

*Ex vivo* rodent lung models are explored for physiological measurements of respiratory function with hyperpolarized (hp) ^129^Xe MRI. It is shown that excised lung models allow for simplification of the technical challenges involved and provide valuable physiological insights that are not feasible using *in vivo* MRI protocols. A custom designed breathing apparatus enables MR images of gas distribution on increasing ventilation volumes of actively inhaled hp ^129^Xe. Straightforward hp ^129^Xe MRI protocols provide residual lung volume (*RV*) data and permit for spatially resolved tracking of small hp ^129^Xe probe volumes during the inhalation cycle. Hp ^129^Xe MRI of lung function in the excised organ demonstrates the persistence of post mortem airway responsiveness to intravenous methacholine challenges. The presented methodology enables physiology of lung function in health and disease without additional regulatory approval requirements and reduces the technical and logistical challenges with hp gas MRI experiments. The post mortem lung functional data can augment histological measurements and should be of interest for drug development studies.

## Introduction

The use of animal models of pulmonary diseases is well established in many areas of biomedical research, however *in vivo* functional respiratory measurements of ventilated and anesthetized small animals are technically challenging to achieve [1]–[3]. Nonetheless, *ex vivo* ventilated lungs have been used as a model to investigate airway responses [4]–[6].

Several investigators have since utilized isolated and perfused rodent lungs to study lung vascular function and to monitor inflammatory responses to noxious stimuli, for example, lipopolysaccharides and prolonged hyperventilation in the absence of systemic interactions [7]–[13]. In addition isolated and perfused murine lungs have been used to investigate pharmacokinetics of inhaled aerosols [14], [15].

Uhlig et al. have performed technically challenging experiments on the intact *ex vivo* murine lungs examining both the airway and the vascular responses to intravenous delivery of a variety of pharmacologically active substances including methacholine, serotonin, endothelin-1 and leukotriene C_4_. [16]. The reported changes in airway resistance and vasoconstriction correlated well with the results obtained from precision cut lung slice models.

### Hyperpolarized Noble Gas Magnetic Resonance Imaging (MRI)

Current imaging techniques provide regional information but suffer from notable difficulties when applied to the pulmonary system. High resolution computed tomography (HRCT) provides high temporal and spatial resolution images but generates limited functional information. Nuclear medicine techniques such as single photon emission computed tomography (SPECT) and positron emission tomography (PET) have provided important additional information [17]–[21] but offer limited temporal and spatial resolution with higher doses of ionizing radiation.

Conventional proton MRI of the lungs aimed at studying lung parenchyma suffers from low sensitivity resulting from the inherently low tissue to volume ratio and local magnetic field inhomogeneities associated with the void space of the lungs. Furthermore, low signal intensities combined with short T_2_^*^ relaxation times place significant limitations on MRI hardware and on MRI protocols resulting in diminished image resolution [22], [23].

Hyperpolarized (hp) noble gas MRI of the lungs increasingly establishes itself as an alternative technique for imaging of the lung airspaces [24], [25]. Due to the large gyromagnetic ratio (γ) of helium-3 (^3^He), its high diffusivity, and its ability to assume high levels of hyperpolarization, hp ^3^He has been extensively used in lung ventilation imaging studies and in characterization of alveolar geometry [26]–[28]. An additional noble gas isotope, xenon-129 (^129^Xe) has attracted increasing attention for hp pulmonary MRI applications partially due to the limited availability of ^3^He but also because of the ability to interrogate additional clinical parameters [29]–[33]. For example, surface to volume ratio in lungs can be probed with hp ^129^Xe because of the high solubility of ^129^Xe in tissue and its wide chemical shift range. The ^129^Xe chemical shift leads to distinguishable MR signals for xenon dissolved in blood, tissue, and xenon in the gas phase, thus enabling regional studies of gas exchange through the parenchyma [32].

Hp ^129^Xe can be produced by spin exchange optical pumping (SEOP) [34], [35] with pulmonary MRI obtained after inhalation of the hp ^129^Xe [24], [33], [36], [37]. As xenon becomes a general anesthetic if inhaled in high concentrations special care is required for its *in vivo* clinical usage [38]. Oxygen can be added to the hp gas for inhalation but the presence of paramagnetic O_2_ leads to the faster destruction of hp xenon state thus limiting the duration of experiments. [39].

In addition to the difficulties of performing MR measurements on anaesthetized and ventilated subjects, not excluding logistical concerns, *in vivo* hp ^129^Xe MRI in small animals requires precision control of hp gas delivery with large amounts of specialist technical hardware such as hp ^129^Xe compatible ventilators and delivery systems [40], [41] further adding to the significant costs involved. Excellent high quality MR images have been reported, although thus far the technique is limited to a few, highly specialized, research centers.

*Ex vivo* lung models in conjunction with hp gas MRI therefore offer the opportunity to reduce the experimental complexity. The use of the *ex vivo* model should facilitate rapid development and testing of hp gas MRI protocols whilst allowing regional study of lung responses in the absence of systemic effects. Furthermore, *ex vivo* pulmonary MRI allows for tests of lung function using protocols, such as prolonged breath holds or the omission of oxygen, that may be beneficial for obtaining certain parameters but that are not feasible for studies with living animals. Finally, *ex vivo* lung models may reduce the severity of the procedure to the experimental animals minimizing regulatory approval requirements, whilst potentially providing a solid platform for rapid drug development and advancement.

## Materials and Methods

### Animal care and preparation

The University of Nottingham Ethical Review Committee approves the study, which is carried out in strict accordance with local animal welfare guidelines and the UK Home Office Animals (Scientific Procedures) Act 1986. All efforts are made to minimize animal suffering.

Healthy male Sprague-Dawley rats (175–300 g, n = 20, Charles River UK Ltd, Margate, UK) and Dunkin Hartley guinea pigs (200–300 g, n = 8, Harlan UK Ltd, Shardlow, UK) are terminated by overdose of pentobarbital (Sigma-Aldrich Ltd, Gillingham, UK). After confirmation of death, surgery is performed postmortem. A catheter is inserted into the right ventricle or caudal vena cava to permit flushing of the pulmonary circulation with heparin-saline solution (Wockhardt UK Ltd, Wrexham, UK) followed by Dublecco's phosphate buffer solution (D-PBS, Sigma-Aldrich Ltd, Gillingham, UK) to remove remaining blood from the pulmonary circulation.

**Figure 1:**
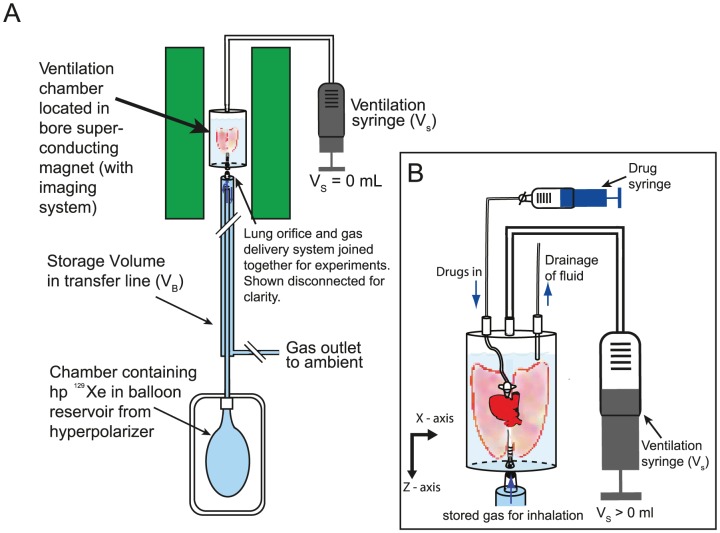
Outline of the hyperpolarized ^129^Xe gas delivery to the *ex vivo* lung. (A) Experimental *ex vivo* setup with hp ^129^Xe administered from a balloon reservoir chamber into the storage volume (*V_B_*) before being inhaled by the lung. The lung is caused to inhale (exhale) by the negative (positive) external ‘pleural’ pressure applied via the suction volume (*V_s_*) from the ventilation syringe upon the artificial pleural cavity; (B) *Ex vivo* lung submerged with its orifice down (sutured to a cannula) in 5% glucose solution within the ventilation chamber with its posterior-anterior axis aligned in z-direction. In this sketch, a negative pleural pressure caused by *V_s_* leads to a partial inflation of the *ex vivo* lung, inhaling a selected gas (hp ^129^Xe, or N_2_ or O_2_) from the storage volume *V_B_*. Drugs are administered via a cannula sited in the right ventricle with the excess fluid outlet located below the fluid level in the chamber. All resulting MR images shown in subsequent figures are depicted with the lung orifice pointing upwards.

The heart and lungs are subsequently removed *en masse*. A plastic adapter tube is placed 5–10 mm above the carina and sutured into place. The heart and lungs are then transferred into a custom-built acrylic ventilation chamber with the lungs suspended in 5% glucose solution (weight/volume) (Baxter Healthcare Ltd, Thetford, UK) to minimize dehydration or swelling of the tissues [42] with the trachea pointing downwards as detailed in Fig. 1. In this situation it is known that there is a pressure gradient of no more than 0.5 kPa (5 cm H_2_O) from the base to the apex of the lung as the fully expanded lung never exceeded 5 cm in length. The *ex vivo* lungs are checked on repeated inflations with 4–5 mL of ambient air for leakage either from the suture around the trachea or the lungs themselves. The lungs are chilled for transportation to the imaging facility with temperature maintained, well above the freezing point, at 278 K. The transfer from the extraction to the experiment facility takes approximately 90 min. After transportation, the lungs are then passively warmed to ambient temperature before imaging experiments.

### Production of hp ^129^Xe

Hp ^129^Xe is produced in batch mode using spin exchange optical pumping (SEOP) [34] of a gas mixture containing 25% Xe (enriched to 83% ^129^Xe, Nova Gas Technologies, Charleston, SC, USA) and 75% N_2_ (99.999% pure, Air Liquide, Coleshill, UK). SEOP is performed at 40 kPa followed by expansion of the hp gas into the evacuated chamber of the hp gas extraction unit [43]. The chamber allows for the recompression of the hp gas to ambient pressure and thus makes it available for inhalation. The technical details of the extraction and compression process are beyond the scope of this paper and will be reported elsewhere [44]. The hp xenon delivered to the excised rodent lung for inhalation is spin polarized to P = 40%. However, taking the four fold dilution with nitrogen in the SEOP gas mixture into account, the apparent spin polarization [43] is P_app_ = (40÷4)% = 10% (i.e. leading to the same signal intensity that would be obtained from pure xenon polarized to P = 10%).

### Ex vivo lung ventilation

Active inhalation of air or hp ^129^Xe inside the magnet is accomplished by a small degree of suction provided by a ventilation syringe that causes the lung to inflate as previously demonstrated with hp ^83^Kr [45], [46]. Briefly, negative pressure is applied to the artificial pleural cavity of the breathing apparatus by creating a desired suction volume *V_s_* within the air filled ventilation syringe shown in Fig. 1. The application of the suction volume *V_s_* typically leads to ‘pleural pressures’ in the artificial pleural cavity around +0.5 to −3 kPa (+5 to −30 cm H_2_O) causing the lungs to inflate and therefore inhale a volume *V_i_*. Due to the use of the compressible fluid (i.e. air) within the ventilation syringe and the tubing, the inhaled volume *V_i_* is not identical to *V_s_* but can be determined experimentally. Following inhalation to *V_i_*, this gas volume is completely exhaled through an increase in the pleural pressure by the reversal of the suction volume to *V_s_* = 0. The exhaled gas is channeled via teflon tubing into a water bell located outside of the magnet. The exhaled gas volume is determined directly by the volume of displaced water. The average *V_i_* values obtained in 3 healthy lungs as a function of the suction volume *V_s_* are listed in Table 1.

**Table 1:** Relationship between syringe suction volume and inhaled gas volume^1^.

*Applied Syringe Volume, V_s_* (mL)	*Corresponding Inhaled Volume, V_i_* (mL)			Average V_i_ (mL)
0.5±0.1	-	-	0.3±0.1	**0.3±0.1**
1.0±0.1	0.3±0.1	0.2±0.1	0.5±0.1	**0.3±0.1**
1.5±0.1	-	0.5±0.1	-	**0.5±0.1**
2.0±0.1	0.5±0.1	1.2±0.1	1.1±0.1	**0.9±0.2**
2.5±0.1	-	1.5±0.1	-	**1.5±0.1**
3.0±0.1	1.4±0.1	2.2±0.1	1.7±0.1	**1.8±0.2**
4.0±0.1	2.2±0.1	3.3±0.1	2.1±0.1	**2.5±0.2**
5.0±0.1	2.9±0.1	3.6±0.1	3.3±0.1	**3.3±0.2**
6.0±0.1	3.9±0.1	5.0±0.1	4.2±0.1	**4.4±0.2**

1 Applied syringe suction volumes, *V_s_*, with corresponding values for inhaled volume, *V_i_*, determined by the water bell method for three Sprague-Dawley rats (weight 250–300 g). Errors listed are experimental relative errors. The omitted values were not determined.

### Ventilation Schemes

Prior to hp gas administration the lungs are purged of oxygen. The transfer line with storage volume *V_B_* (Fig. 1a) is flushed with N_2_ (99.999% pure, Air Liquide, Coleshill, UK) and the lungs are ventilated 8–10 times with N_2_ to remove any residual O_2_. The hp gas is then delivered into the storage volume *V_B_* and a suction created through *V_s_* is applied to the artificial pleural cavity causing the lungs to inhale the hp gas. The maximal *V_s_* applied to create suction was 5–6 mL during all experiments, equating to an inhalation volume *V_i_* of 4–5 mL depending on the *ex vivo* lung as detailed in Table 1. In order to target specific regions of the lung, gas is inhaled at different stages of the ventilation cycle. For instance, a small amount of the hp gas is inhaled at the start of the inhalation followed by ‘dark’ (non hp) gas, usually N_2_, or a small volume of hp gas is inhaled at the end of the inhalation following the initial dark gas inhalation to localize the gas to different regions of the lung.

### Bronchoconstriction and reversal

Animals used for airway responsiveness experiments have the catheter used for flushing of the pulmonary circulation retained with the cranial and caudal vena cava ligated to ensure drug delivery to the pulmonary circulation. The cannula is sutured into place and attached to a fine perfluoroalkoxy (PFA) tube passed through a modified ventilation chamber as detailed in Fig. 1b) with the drug syringe located outside the superconducting magnet.

In order to satisfy tissue metabolic demands, the storage volume *V_B_* is flushed with 50 mL O_2_ prior to hp gas delivery whilst the lungs are ventilated 8–10 times with the oxygen. This is followed by purging the transfer line with N_2_ prior to hp ^129^Xe delivery as described above.

Bronchoconstriction is achieved by injecting methacholine (Sigma-Aldrich Ltd, Gillingham, UK) through the pulmonary circulation. For rat and guinea pig lungs, 60 µg and 10 µg of methacholine dissolved in 1 mL 0.9% saline solution (Baxter Healthcare Ltd, Thetford, UK) are used respectively. The methacholine solutions are delivered using the drug cannula at a rate of 1 mL/minute and are followed by a 2–3 mL bolus of 5% glucose solution over 2–3 minutes to ensure complete drug delivery through the pulmonary circulation. The reversal is produced by flushing the challenged lungs with 5–10 mL 5% glucose solution and 1000 µg of salbutamol (Allen and Hanbury's Ltd, Middlesex, UK) dissolved in 1.0 mL of 0.9% saline solution over 6–11 minutes with the lungs from both species of animal.

### Pulmonary MRI

Imaging experiments are performed using a 9.4 T vertical bore Bruker® Avance III microimaging system (Bruker Corporation, Billerica, Massachusetts, USA). A custom-built 25 mm low-pass birdcage volume coil tuned to the resonance frequency of ^129^Xe gas in the lung of 110.69 MHz is used in all experiments. Spectroscopic data are collected using experimental schemes discussed in the Results using 3^0^ hard pulses of 4.47 µs at 53 W. Images are acquired using a modified variable flip angle (VFA) FLASH gradient echo pulse sequence [47]. Hard pulses of 134 µs and sinc-shaped pulses of 1000 µs at variable power levels are used for non-slice-selective and slice-selective image acquisition. An individual phase increment is recorded during 2.61 ms; subsequent phase increment acquisitions are separated by 214.5 ms. Therefore the total acquisition time for an image with 128×64 resolution is 13.8 s. All coronal images are acquired in 128×64 image matrices with field of view (FOV) of 46.9 mm and 30.0 mm in the superior and inferior direction, respectively. Slice thickness in slice-selective imaging experiments is 4 mm and the slice-selective frequency offset corresponds to the excitation of the central slice.

### Image processing and analysis

Raw data are analyzed using Prospa© (v. 3.06, Magritek, Wellington, New Zealand) where a sine-bell squared function is used to window the data in both dimensions to result in magnitude images with increased signal to noise ratio. The images are further processed using IGOR Pro© (Wavemetrics, Lake Oswego, Oregon, USA) as follows. A threshold procedure is applied to remove the background noise. To achieve this, the lower threshold is derived from the mean signal intensity plus two standard deviations obtained from a 10×10 voxel region randomly selected outside the lung region within the image limits [48], [49]. This value is subtracted from the intensity in each pixel of the image resulting in reduced noise images. The signal to noise ratio (SNR) with the threshold procedure typically improves by a factor of four from ∼60 to ∼240. Subsequent image analysis is also performed with IGOR Pro©.

## Results

### Measurement of ex vivo lung residual volume

Hp ^129^Xe MRI and NMR (Nuclear Magnetic Resonance) spectroscopy of excised lungs can be used straightforwardly to measure residual volume (*RV*) of excised lungs. The most basic hp ^129^Xe protocol that can be used for RV determination, using non slice selective and non-spatially resolved 1D NMR spectroscopic measurements is described by Eq. 1: 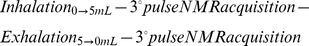
(1)


Upon inhalation, the hp gas will be diluted by the gas in the residual volume *RV* (i.e. N_2_ or thermally polarized, MRI non-detectable xenon with N_2_) to an unknown hp gas concentration with total volume *V_i_+RV*. The residual volume, as defined in this paper, is composed of the alveolar residual volume and, to a lesser extent, the anatomic dead space in the ‘conducting zone’. The hp gas concentration will remain unchanged during exhalation. Therefore the difference between the signal intensities found between inhalation and exhalation is caused only by the difference in the respective hp gas volumes in the lung and is not affected by the gas mixture. The signal change relates directly to the ratio of gas volume in the inhaled lung to the residual volume *RV*:
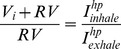
(2)where 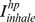 is the NMR signal intensity recorded with a 3° pulse from a lung with the gas phase volume *V_i_+RV* after inhalation of a *V_i_* volume of hp gas and 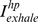 is the signal intensity from a 3° pulse on exhalation to *RV*.

In order to obtain the residual volume *RV*, the inhaled volume *V_i_* for each lung is determined at a constant suction volume *V_s_* = 5 mL (i.e. 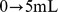) using the water displacement method as described in the experimental section. The residual volume can then be calculated using:
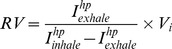
(3)


The *RV* values obtained for three different, but similar sized, rat lungs are shown in Table 2. Please note the underlying assumption is that the lung is ventilated without areas affected by ventilation defects (i.e. non-ventilated lung regions) as further elaborated on in the Discussion section.

**Table 2:** Experimentally determined *ex vivo* lung residual volumes (*RV*)^1^.

*Rat Weight (g)*	*Inhaled Gas Volume, V_i_ (mL)*	*Calculated Residual Volume, RV (mL) spectroscopy*	*Calculated Residual Volume, RV (mL) VFA FLASH images*
276	3.65±0.10	1.03±0.08	1.04±0.09
286	3.58±0.10	1.03±0.04	1.07±0.10
266	3.58±0.15	1.22±0.03	0.91±0.07
**276**	**3.60±0.06**	**1.09±0.03**	**1.01±0.04**

1 The *ex vivo* lung residual volume, *RV*, calculated using inhaled volume *V_i_* values determined experimentally for the suction volume *V_s_* = 5.0 mL with respective standard errors (n = 4). The *RV* values are derived with standard errors (n = 4) from non-spatially resolved spectroscopic measurements (Eq. 4) and from the non-slice selective coronal VFA FLASH imaging sequence (Eq. 5) are also shown for comparison.

The second scheme uses spatially resolved experiments in order to determine the uniformity of ventilation. In this scheme the total MRI signal obtained after full inhalation of hp ^129^Xe (i.e. *V_s_* = 5 mL, see Eq. 4) is compared with the signal of a second MRI scan obtained in a separate experiment using full inhalation of hp ^129^Xe followed by immediate complete exhalation (Eq. 5). 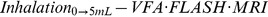
(4)

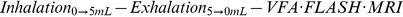
(5)


**Figure 2:**
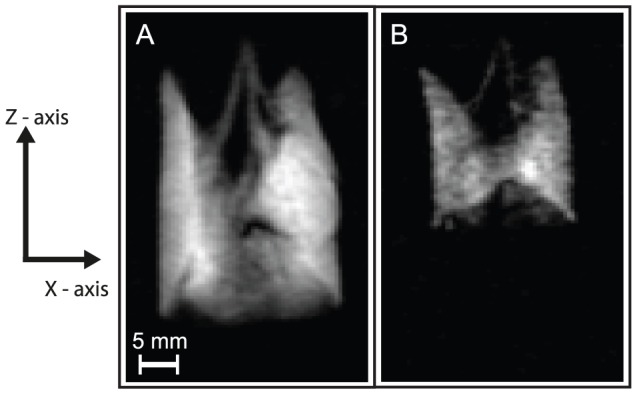
Non-slice selective coronal VFA FLASH MR images used for calculation of residual volume (*RV*). (A) Acquired after inhalation to *V_s_* = 5 mL (actual inhalation, *V_i_* = 3.09 mL); (B) Inhalation to *V_s_* = 5 mL followed by full exhalation to *V_s_* = 0 mL (*V_i_* = 0 mL) before the MR image is acquired. Image resolution is 128×64 with FOV = 46.9 mm in the longitudinal and FOV = 30.0 mm in the axial dimensions, respectively. In this presentation, the orifice of the lung is pointing up with the posterior-anterior axis aligned with the z-direction.



Coronal non-slice selective VFA FLASH imaging sequences are used in both cases and Fig. 2 displays the resulting images. The residual volume can be determined using the total MRI signal intensity resulting from the sum of intensities from each of the *n*×*m* volume elements (voxels) according to:

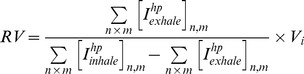
(6)where (*n, m*) is the voxel index, 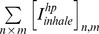 and 
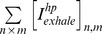
 are the summated voxel intensities on inhale and exhale respectively.

Since each VFA Flash MRI sequence uses – and therefore destroys – the complete hyperpolarization to record the image, the two MR images for inhalation and inhalation with exhalation need to be acquired in two separate experiments with separate hp gas deliveries. As a consequence, these measurements may be complicated by fluctuations in the SEOP process leading to a scatter in the obtained hyperpolarization levels. Therefore, the two MR images require a normalization that can be readily accomplished by recording a small flip angle pulse NMR spectrum for calibration purposes after the initial inhalation (*V_s_* = 5 mL) in both experiments as shown below: 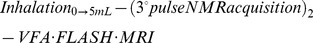
(7)

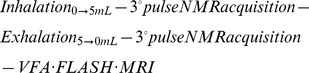
(8)


Note that the sequence in Eq. 8 also contains a second small flip angle pulse (after exhalation) that is used for the additional *RV* determination through the non-spatially resolved (spectroscopic) scheme described in Eq. 1. As an additional refinement, Eq. 7 contains a second 3° pulse – NMR acquisition step after inhalation to ensure that the spin polarization is similarly depleted by an identical number of 3° pulses in schemes 7 and 8. Values for *RV* obtained through both schemes (i.e. spectroscopic and through MRI) are displayed in Table 2 for three rat lungs with an average value of *RV* = 1.1±0.1 mL and *RV* = 1.0±0.1 mL using the NMR spectroscopic and MRI methods respectively.

### Studying lung ventilation as a function of inhalation volume *V_i_*

**Figure 3:**
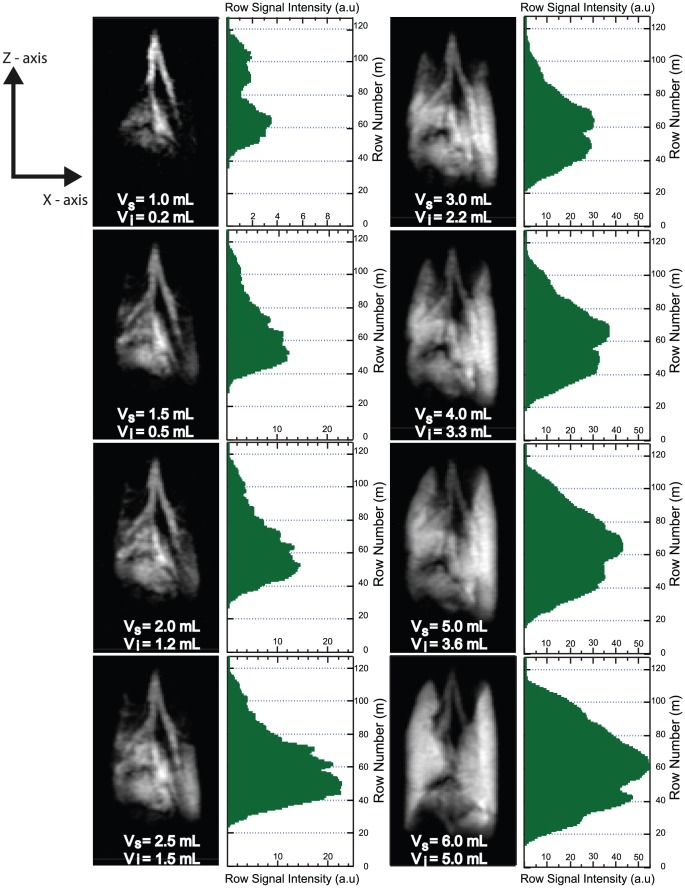
Hyperpolarized ^129^Xe gas distribution on increasing inhalation volumes. Non-slice selective coronal VFA FLASH images as a function of increasing suction volume (*V_s_*) (and inhaled volume (*V_i_*)). The corresponding histograms displaying integrated intensities, 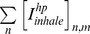, for each row, *m*, are shown to the right of the images. The vertical axis of the image is parallel to the direction of the *B_o_* field (z-direction) and corresponds to the posterior-anterior axis (base to apex) of the lung in the magnet. Phase encoding is applied transverse to the *B_o_* field direction. As the suction volume increases from 0.5 mL to 6.0 mL the image contrast is greatly enhanced. The effect is caused by the increasing quantities of inhaled hp gas contained in the lung as the suction volume rises. Matrix 128×64 with FOV = 46.9×30.0 mm^2^.

The *ex vivo* lung imaging apparatus described in Fig. 1 allows for a large range of ventilation volumes, *V_i_*, to be used for pulmonary hp ^129^Xe MRI. These experiments can provide insights into how lungs are ventilated regionally as the *ex vivo* model permits ‘freezing’ of ventilation to take the MR images at various points of the ventilation cycle. In this work we use lungs from similarly sized and healthy Sprague Dawley rats. Non-slice selective coronal MRI images displayed in Fig. 3 are acquired as the inhalation volume *V_i_* is increased from 0.3 ml to 5.0 mL (i.e. with the suction volume *V_s_* ranging from 1.0 ml to 6.0 mL). The corresponding integrated intensities 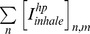 for each of the *m* rows are shown to the right of the MR images in Fig. 3. The histograms are obtained using a voxel counting algorithm where all voxels across each row are added to give a measure of ventilation as longitudinal position (i.e. along the z axis) from the base to the end of the trachea (Fig. 3).

**Figure 4:**
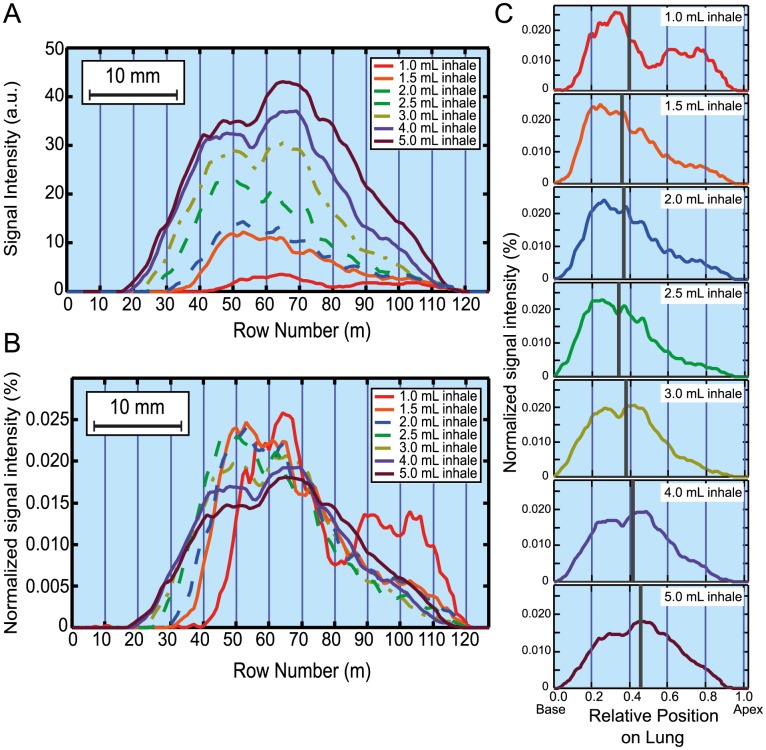
Normalization of hyperpolarized ^129^Xe distribution by total signal intensity and position along the anterior-posterior axis. (A) Integrated signal intensity (taken from Fig. 3) in arbitrary units (a.u.) as a function of the image row number *m* (in z-direction); (B) Integrated signal intensity after normalization by the total signal intensity (i.e. the integrated intensity of all voxels, 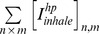, of the respective MR image); (C) Normalized integrated signal intensity as in (B) but as a function of position along the lung posterior-anterior axis (z-axis) from base to apices. Independent of inhalation volume and actual lung expansion, the 0.0 point refers the base of the lung, whereas 1.0 refers to the apices. The 50% signal intensity position in the lungs is indicated by grey vertical line (C) i.e. 50% of the total signal intensity lies to both sides of the grey line.

As can be seen from the histograms in Fig. 3, at *V_s_* = 1.0 mL the initial region of lung inflation is largely located in the base of the lung with the majority of the signal resulting from either the base or the major conducting airways. As the base expands between *V_s_* = 1.0–2.5 mL the further drop in negative pleural pressure causes adjacent lung regions to inflate and the apices start to display significant inflation at *V_s_*>2.5 mL. Further inflation increases lung length with signal intensity growing across all lung regions. To better illustrate the inhalation physiology, the histograms of Fig. 3 are further processed and presented in a slightly different format in Fig. 4.

It is instructional to normalize each histogram from Fig. 3 by the total signal intensity arising from the lung after inhalation of the volume *V_i_* of hp gas, 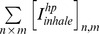, to allow for better comparison of the regional gas distribution between the various inhalation volumes *V_s_* as shown in Fig. 4b). In Fig. 4c) a further normalization has been performed on the data where the x-axis (row number in the histograms in Fig. 4b)) is divided by the length of the expanding lung to reveal the relative position within the lung. Normalized intensities as a function of relative position within the lung allow for a better visualization of the regional differences in ventilation as the total inhalation volume *V_i_* is changed. Initially at low suction volume *V_s_* = 1.0 mL (*V_i_* = 0.2 mL), it is seen that that the largest portion of the MR signal originates from the base of the lung with a smaller contribution from the larger conducting airways. On increasing inhalation the base receives a growing share of the signal until at *V_s_* = 2.5 mL (*V_i_* = 1.2 mL) the distribution begins to shift from the base towards the apices. The grey line in Fig. 4c) indicates the position with equal integrated intensity on both sides of this position. This 50% signal intensity position marker serves as an additional aid to visualize regional ventilation of the lung. Initially, this line shifts towards the base of the lung as the suction volume is increased up to *V_s_* = 2.5 mL. This shift reflects the placement of the inhaled gas predominantly into the lung base. With further increasing inhalation causing increasing ventilation of the apices, the line shifts into the opposite direction and at *V_s_* = 5.0 mL (*V_i_* = 3.6 mL) it is centered approximately at the midpoint of the lung.

### Timed release of a small quantity of hp ^129^Xe during constant volume *V_i_* inhalation

**Figure 5:**
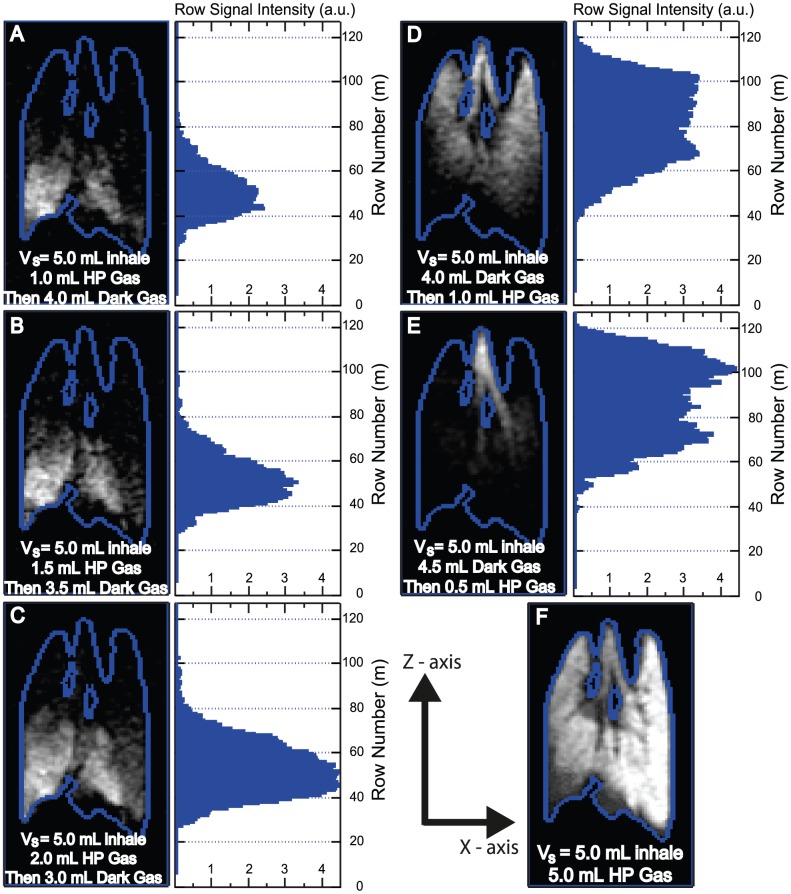
Timed release of hyperpolarized ^129^Xe during constant inhalation volumes. Coronal slice selective VFA FLASH images for directed ventilation schemes with a histogram that displays the integrated intensities in each row are shown to the right of the images. Scheme 1 (A–C)- initial inhalation consists of a known volume of hp gas, *V_s(hp)_*, followed by dark gas, *V_s(Dark)_*. Scheme 2 (D–E)- the reversal with the inhalation of *V_s(Dark)_* followed by *V_s(hp)_*. Full 5.0 mL inhalation of hp gas with edge detection using Kirsch operator [71] with window level adjusted to show lower signal intensities (F). Z-axis along *B_o_* in posterior-anterior axis (base to apex) of the lung in the magnet and x-axis along indirect (phase encoding) dimension. Imaging parameters: 4 mm central slice, matrix 128×64, FOV = 46.9×30.0 mm^2^. Positioning of the lung as in Fig. 2.

*Ex vivo* pulmonary ^129^Xe MRI also allows for the timed release of a small bolus (0.5–1.0 mL) of hp gas during the inhalation period. This method provides further data to support the assertion that the initially inhaled gas localizes to the base of the lung and is directed towards the apices mostly at the end of the inhalation. Two inflation schemes with a total suction volume of *V_s_* = 5.0 mL are employed. For scheme 1 in Fig. 5a–c)- the initial inhalation consists of a chosen fraction of hp gas, inhaled through application of suction volume *V_s(hp)_*, followed by ‘dark’ (i.e. MRI inactive, usually N_2_) gas. The dark gas is inhaled after flushing of the storage volume *V_B_* with N_2_ and applying suction volume *V_s(dark)_*. In scheme 2 in Fig. 5d–e) the delivery order is reversed with the initial dark gas delivery using N_2_ and suction volume being *V_s(dark)_* followed by hp ^129^Xe delivery into *V_B_* and suction volume *V_s(hp)_*. Using ventilation scheme 1 with *V_s(hp)_* = 1.0 mL followed by *V_s(dark)_* = 4.0 mL, the MRI shows that the hp gas signal is directed to the base. As the ratio *V_s(hp)_*/*V_s(dark)_* increases, at constant *V_s(hp)_*+*V_s(dark)_* = *V_s_* = 5.0 mL, the hp gas is progressively found further towards the apices. In scheme 2 the hp gas is directed more to the apical regions of the lung with the hp gas seen in the larger conducting airways. Further increase of the dark gas component in scheme 2 (Fig. 5e)) results in the hp gas being localized to the conducting airways themselves.

### Airway Responsiveness

**Figure 6:**
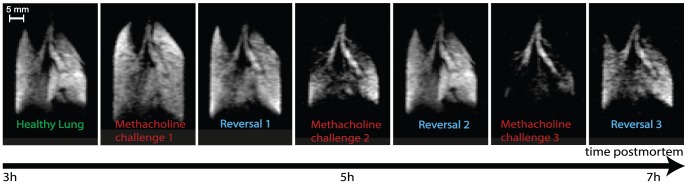
Airway responsiveness testing in an excised rat lung. Slice selective VFA FLASH images of positively responding *ex vivo* rat lungs after intravenous challenges of 60 µg methacholine with subsequent reversal produced by flushes of intravenous 5% glucose and 1000 µg salbutamol. Images were performed using a constant inhalation syringe (suction) volume of *V_S_* = 5 mL. Imaging parameters: 4 mm central slice, matrix 128×64, FOV = 46.9×30.0 mm^2^. Positioning of the lung as in Fig. 2.

Excised lung tissue, including lung slices for optical microscopy, has regularly been used to study airway responsiveness to challenges with bronchial smooth muscle agonists such as methacholine (MCh) [50]–[52]. In this work, it is investigated whether the whole organ can be used many hours post mortem for pulmonary hp ^129^Xe MRI of MCh challenges. Furthermore, the possibility of the reversal of airway responsiveness by flushing the pulmonary circulation with glucose and salbutamol solutions followed by subsequent challenges and reversals are also explored. Images obtained from rat lungs, positively responding to MCh challenges, are shown in Fig. 6. Initially it can be seen on the first MCh challenge that the lung hyperinflates due to gas trapping with increasing inflation of other pulmonary units if the suction volume *V_s_* is kept constant. The hyperinflation then recovered on reversal with flushing the lung with glucose and salbutamol. A subsequent, second challenge produces significant ventilation defects. After reversal, the third challenge causes the majority of lung tissue to fail to receive hp gas due to the severity of the bronchoconstriction. Nevertheless, these severe effects, that would have likely caused termination of any *in vivo* experiment, could still be partially reversed again.

A very similar response is demonstrated on three further occasions but subsequent rat lungs (seven in total) showed little or no response to MCh at the dosages under investigation (data not shown). Note that the Sprague Dawley rats are healthy and have not been not sensitized to display any airway hyper-responsiveness. The purpose of this proof of principle study is not to explore airway responsiveness in detail but to demonstrate that responsiveness, if present, can be triggered, observed and reversed for several hours post mortem.

**Figure 7:**
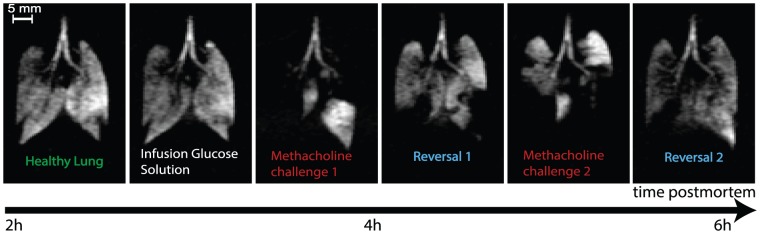
Airway responsiveness testing in an excised guinea pig lung. Slice-selective VFA FLASH images of *ex vivo* guinea pig lungs after intravenous challenges with 5% glucose solution alone and 10 µg methacholine. Subsequent reversal was produced by flushes of intravenous 5% glucose and 200 µg salbutamol. Images were performed with a constant inhalation syringe (suction) volume of *V_S_* = 5 mL. Imaging parameters: 4 mm central slice, matrix 128×64, FOV = 46.9×30.0 mm^2^. Positioning of the lung as in Fig. 2.

Rat lungs are compared to guinea pig lungs as the latter are known to have greater quantities of bronchial smooth muscle [53], [54]. Similar patterns of ventilation defects are produced by smaller dosages of MCh on the three lungs imaged with these again found to be partially reversible with glucose and salbutamol flushes allowing further challenges for several hours post mortem as demonstrated in Fig. 7. It is however noted that reversal of the ventilation defects in guinea pig lungs depends more on flushing of residual MCh from the pulmonary circulation rather than significant improvements with salbutamol.

## Discussion

### The residual volume, RV, of ex vivo lungs

Residual volume, *RV*, is an important functional parameter used in both animal models of pulmonary disease and in the clinical setting. *RV* is found to decrease in patients with restrictive lung diseases such as fibrotic lung disease and rises in patients with obstructive disease due to hyperinflation. Many methods have been utilized for measurement of *RV* in small animals [1]. In this work, the calculated value of the residual volume of 1.1±0.1 mL using the MR spectroscopic measurements and 1.0±0.1 mL using the spatially resolved MRI method agree within the experimental error. The values are however lower than the 1.26 mL that was previously determined using body plethysmography [55] and of ∼1.6 mL with neon dilution [56] for similar sized rats. In the *ex vivo* rodent lung at *V_s_* = 0 mL it is likely that this situation is more akin to an open-chested animal where there is no chest wall recoil holding the airways open with the result that the calculated value of *RV* will be reduced as has been noticed in dog lungs [57]. A further, small contribution to the difference found between our value and previous data is caused by the shortening of the conducting airways (and hence a shorting of the anatomic dead space) as the cannula was sited just above the carina rather than higher below the larynx in the *in vivo* experiments. On the other hand, it is known that lung compliance decreases with temperature [58] with the result that as the lungs are kept at ambient temperature or just below this will compensate for some of the aforementioned reduction in *RV*. Finally, it has been noted by several groups that gas trapping is an issue with excised lung tissue used for ventilation studies [4], [59], [60]. Gas trapping has not been a noticeable feature in the current study as no significant gas trapping is seen with prolonged lung ventilation. It its unknown whether the lack of gas trapping is due to differences in the method of organ preparation, smaller total inhaled volume in the current work, or the different rat strain used (Sprague-Dawley in the current study). Note that significant gas trapping is seen with some of the guinea pig lungs causing them to be rejected for imaging.

The presented method is a fast and straightforward addition to hp gas MRI of excised lungs requiring no additional instrumentation. Furthermore, the spatially resolved 2D method could also be modified to reduce the contribution from the signal of the airways (i.e. the anatomical dead space) to the residual volume determination. Some airway contribution to the MRI signal can be taken directly from the images in Fig. 2a–b) or could be measured in more detail for example through a directed ventilation scheme as in Fig. 5e). The directed ventilation scheme can in principle also provide information about regional contributions to the residual volume. Note however, that the underlying assumption made for the *RV* determination in this work is that the hp gas mixes with the ‘dark’ gas in the residual lung volume uniformly. This requires, that the lungs are being inhaled with hp gas without areas of restricted or obstructed ventilation. Deviations form the expected *RV* in healthy lungs would be indicative of the presence of pulmonary diseases. However this was not further investigated as animal models of disease are beyond the scope of this work.

Measuring functional respiratory parameters, such as *RV in vivo* using hp gas imaging experiments in rodents has proven difficult due to the small gas volumes. The schemes to calculate *RV* developed with the *ex vivo* model in this work may provide a valuable addition to physiological methodology. As an alternative to existing lung function tests, the *ex vivo* hp ^129^Xe MRI method provides spatially resolved information of the distribution of the *RV* which might provide a sensitive test to identify regions disproportionately affected by the disease process. The hp ^129^Xe MRI method detailed here, being exceptionally simple, could easily be translated to *in vivo* MRI in the preclinical or clinical setting.

### Ventilation physiology using ex vivo lungs

Image data on increasing ventilation volume presented in this work potentially provide new insights into pulmonary physiology. It is shown that ventilation in the *ex vivo* models produces initial ventilation from the bases of the inverted lungs increasing downward towards the apices. Whether this is due to an inherent property of the lung or some element of the experimental set up, for instance, with the lungs submerged in glucose solution with a small pressure gradient of <0.5 kPa (5 cm H_2_O) along the length of the lung, is as yet unknown.

Classical pulmonary physiological theory has tended to explain differences in regional ventilation in humans due to the gravitational effects on pleural pressure resulting in the lower, most dependent, lung regions being under higher resting pressure and hence on inhalation receive higher volumes of gas [61], [62].

Previous works with SPECT and xenon enhanced CT have shown regional differences in ventilation and changes due to posture in animals [17], [63], [64]. Marcucci et al., using xenon enhanced CT, studied the vertical ventral/dorsal (V/D) ventilation gradient in supine canines where it was noted that the dorsal lung receives the greatest ventilation in the supine position [65]. This gradient was abolished once the animals were placed in the prone position. Interestingly, the group also found a ventilation gradient between the base of the lung and the apex (anterior–posterior) where the base experiences higher levels of ventilation compared to the apex with the animal supine, although this was removed with the animal prone.

Månsson et al. studied V/D fractional ventilation gradients *in vivo* using hp ^3^He MRI in rats noting a similar V/D gradient in the supine position and reporting the removal of the gradient with the animal prone [66]. Couch et al. subsequently confirmed this in rats using both hp ^3^He and hp ^129^Xe, also noting a small positive posterior/anterior (base to apex) fractional ventilation gradient in this work [67].

However recently Kyriazis et al. have noted that in a rat elastase model of emphysema ventilated by positive pressure and imaged using hp ^3^He, inflation rates at the bases reduced more than at the apices compared to controls despite apparent diffusion coefficients (markers of emphysematous damage) indicating changes throughout the lung [68]. It is therefore possible that this was due to some inherent elastic property of the lung indicating underlying regional differences.

In this work, the experimental arrangement provides further evidence that regional differences in ventilation may be due to inherent elastic properties of the lung as at values of inhaled volume close to those studied elsewhere (1–2 mL in this size of rats), most of the inhaled gas localizes to the bases even when these regions are most superior. Further work to confirm this would be required to see if this situation changed once the lungs were suspended from the trachea without the lungs submerged in solution (i.e. trachea most superior) and also if the lungs were, in supine or prone position as has been performed by other groups with excised lung tissue [59].

### Whole organ response to post mortem MCh challenges

The image data presented in this work confirms post mortem airway responsiveness to MCh challenges and glucose/salbutamol reversal in *ex vivo* rat and guinea pig lungs. Not all of the healthy *ex vivo* rat lungs respond to MCh challenge but those that do respond show regional ventilation defects at drug dosages similar to those reported elsewhere with a significant increase in sensitivity to methacholine in guinea pig lung tissue. The variation in the airway responsiveness of rat lungs has been documented with review of the literature revealing that there is significant variation in response to MCh amongst rats, especially out-bred strains, when recorded with body plethysmography often requiring very large dosages of MCh [54], [69], [70]. Guinea pig lungs were studied as they are known to have higher levels of bronchial smooth muscle and so are more responsive to MCh [53], [54]. The results confirm this increased airway responsiveness with lungs showing large degrees of bronchoconstriction with one sixth of the dosage. Reversal however appeared to be unaffected by salbutamol and relied more on flushing methacholine from the pulmonary circulation.

The severity of observed bronchoconstriction in some cases in this work is unlikely to be recorded *in vivo* due to the significant physiological deterioration that would result (likely resulting in death before imaging). Therefore the *ex vivo* model offers the opportunity to explore the most extreme of pathophysiological situations for prolonged periods of time in the absence of systemic effects and considerations. The current model could be further improved to incorporate recirculation of fluid and the use of bubble traps to prevent gas emboli in the lung vasculature [10]. With such improvements and the use of more physiological perfusate the model might be able to last beyond the currently reported 7–8 hours.

### Spatial resolution of Ex vivo hp ^129^Xe MRI

As a final technical note, the *ex vivo* model may potentially allow for higher resolution of the MR images compared to *in vivo* hp ^129^Xe MRI that typically relies on signal averaging over multiple breaths. All MR images presented in this proof of concept work are acquired in a single scan without motional artifacts and provide sufficient image spatial resolution using hp ^129^Xe with an apparent polarization of P_app_ = 10%.

## Conclusions

The aim of this work is to demonstrate the utility of the *ex vivo* pulmonary model for hp ^129^Xe MRI studies. The pulmonary *ex vivo* model offers a nimble platform for developing and testing novel hp gas MRI protocols before translation of the methods for preclinical *in vivo* studies and ultimately into clinical research. The usage of *ex vivo* whole organs also reduces the regulatory requirements for animal care, handling and monitoring for hp gas MRI experiments. In addition, the ability to investigate lung function, for example in the absence of oxygen and by precise control and freezing of the ventilation cycle, demonstrates that *ex vivo* models offer a new investigative tool for lung physiology in their own right. The imaging of dynamic changes in *ex vivo* whole organ may be of interest for drug development studies or as an additional technique to elucidate airway responses in the absence of systemic effects or considerations, allowing the study of extreme pathophysiology.

## References

[1] Oneil JJ, Raub JA (1984) Pulmonary-Function Testing in Small Laboratory Mammals. Environmental Health Perspectives 56: 11–22. 6434299 10.1289/ehp.845611 PMC1568219

[2] Bates JH, Irvin CG (2003) Measuring lung function in mice: the phenotyping uncertainty principle. J Appl Physiol 94: 1297–1306. 12626466 10.1152/japplphysiol.00706.2002

[3] Hoymann HG (2007) Invasive and noninvasive lung function measurements in rodents. J Pharmacol Toxicol Methods 55: 16–26. 16793289 10.1016/j.vascn.2006.04.006

[4] Frazer DG, Weber KC (1976) Trapped Air in Ventilated Excised Rat Lungs. Journal of Applied Physiology 40: 915–922. 931931 10.1152/jappl.1976.40.6.915

[5] Greenwald SE, Collino CE, Berry CL (1988) Invitro Determination of Lung Airway Compliance in Small Animals. Medical & Biological Engineering & Computing 26: 497–502. 3256739 10.1007/BF02441917

[6] Struhar D, Harbeck RJ (1990) An Apparatus for the Measurement of Lung-Volume and Compliance in Mice. Laboratory Animals 24: 328–331. 2270042 10.1258/002367790780865930

[7] Herget J, Chovanec M (2010) Isolated perfused murine lung: A well characterized preparation for studying lung vascular function. Drug Discovery Today: Disease Models 7: 131–135.

[8] Herget J, Mcmurtry IF (1985) Effects of Ouabain, Low K+, and Aldosterone on Hypoxic Pressor Reactivity of Rat Lungs. American Journal of Physiology 248: H55–H60. 3970175 10.1152/ajpheart.1985.248.1.H55

[9] Herget J, Mcmurtry IF (1987) Dexamethasone Potentiates Hypoxic Vasoconstriction in Salt Solution-Perfused Rat Lungs. American Journal of Physiology 253: H574–H581. 3631295 10.1152/ajpheart.1987.253.3.H574

[10] Uhlig S, Wollin L (1994) An Improved Setup for the Isolated-Perfused Rat Lung. Journal of Pharmacological and Toxicological Methods 31: 85–94. 8032099 10.1016/1056-8719(94)90047-7

[11] von Bethmann AN, Brasch F, Nusing R, Vogt K, Volk HD, et al (1998) Hyperventilation induces release of cytokines from perfused mouse lung. American Journal of Respiratory and Critical Care Medicine 157: 263–272. 9445308 10.1164/ajrccm.157.1.9608052

[12] Barrenschee M, Lex D, Uhlig S (2010) Effects of the TLR2 agonists MALP-2 and Pam3Cys in isolated mouse lungs. PLoS One 5: e13889. 21124967 10.1371/journal.pone.0013889 PMC2987752

[13] Siegl S, Uhlig S (2012) Using the one-lung method to link p38 to pro-inflammatory gene expression during overventilation in C57BL/6 and BALB/c mice. PLoS One 7: e41464. 22848503 10.1371/journal.pone.0041464 PMC3404097

[14] Ewing P, Eirefelt SJ, Andersson P, Blomgren A, Ryrfeldt A, et al (2008) Short inhalation exposures of the isolated and perfused rat lung to respirable dry particle aerosols; The detailed pharmacokinetics of budesonide, formoterol, and terbutaline. Journal of Aerosol Medicine and Pulmonary Drug Delivery 21: 169–180. 18518793 10.1089/jamp.2007.0654

[15] Selg E, Ewing P, Acevedo F, Sjoberg CO, Ryrfeldt A, et al.. (2012) Dry Powder Inhalation Exposures of the Endotracheally Intubated Rat Lung, Ex Vivo and In Vivo: The Pulmonary Pharmacokinetics of Fluticasone Furoate. J Aerosol Med Pulm Drug Deliv. 10.1089/jamp.2012.0971 23094685

[16] Martin C, Held HD, Uhlig S (1999) Characterization of pulmonary responses in mice: Comparison of lung slices and perfused lung. American Journal of Respiratory and Critical Care Medicine 159: A871–A871.

[17] Orphanidou D, Hughes JMB, Myers MJ, Alsuhali AR, Henderson B (1986) Tomography of Regional Ventilation and Perfusion Using Krypton 81 m in Normal Subjects and Asthmatic-Patients. Thorax 41: 542–551. 3491441 10.1136/thx.41.7.542 PMC460388

[18] Nagao M, Murase K, Ichiki T, Sakai S, Yasuhara Y, et al (2000) Quantitative analysis of technegas SPECT: Evaluation of regional severity of emphysema. Journal of Nuclear Medicine 41: 590–595. 10768557

[19] Harris RS, Schuster DP (2007) Visualizing lung function with positron emission tomography. Journal of Applied Physiology 102: 448–458. 17038490 10.1152/japplphysiol.00763.2006

[20] West JB, Matthews CM, Holland RAB, Dollery CT (1962) Interpretation of Radioactive Gas Clearance Rates in Lung. Journal of Applied Physiology 17: : 14–&. 10.1152/jappl.1962.17.1.14 14006302

[21] West JB (1962) Regional Differences in Gas Exchange in the Lung of Erect Man. Journal of Applied Physiology 17: 893–898. 14000094 10.1152/jappl.1962.17.6.893

[22] Su S, Saunders JK, Smith ICP (1995) Resolving Anatomical Details in Lung Parenchyma: Theory and Experiment for a Structurally and Magnetically Inhomogeneous Lung Imaging Model. Magnetic Resonance in Medicine 33: 760–765. 7651111 10.1002/mrm.1910330604

[23] Puderbach M, Hintze C, Ley S, Eichinger M, Kauczor HU, et al (2007) MR imaging of the chest: A practical approach at 1.5 T. European Journal of Radiology 64: 345–355. 17900843 10.1016/j.ejrad.2007.08.009

[24] Albert MS, Cates GD, Driehuys B, Happer W, Saam B, et al (1994) Biological Magnetic Resonance Imaging Using Laser Polarized Xe-129. Nature 370: 199–201. 8028666 10.1038/370199a0

[25] Middleton H, Black RD, Saam B, Cates GD, Cofer GP, et al (1995) Mr-Imaging with Hyperpolarized He-3 Gas. Magnetic Resonance in Medicine 33: 271–275. 7707920 10.1002/mrm.1910330219

[26] Altes TA, Powers PL, Knight-Scott J, Rakes G, Platts-Mills TAE, et al (2001) Hyperpolarized He-3 MR lung ventilation imaging in asthmatics: Preliminary findings. Journal of Magnetic Resonance Imaging 13: 378–384. 11241810 10.1002/jmri.1054

[27] Fain SB, Korosec FR, Holmes JH, O'Halloran R, Sorkness RL, et al (2007) Functional lung imaging using hyperpolarized gas MRI. Journal of Magnetic Resonance Imaging 25: 910–923. 17410561 10.1002/jmri.20876

[28] Fain S, Schiebler ML, McCormack DG, Parraga G (2010) Imaging of Lung Function Using Hyperpolarized Helium-3 Magnetic Resonance Imaging: Review of Current and Emerging Translational Methods and Applications. Journal of Magnetic Resonance Imaging 32: 1398–1408. 21105144 10.1002/jmri.22375 PMC3058806

[29] Sakai K, Bilek AM, Oteiza E, Walsworth RL, Balamore D, et al (1996) Temporal dynamics of hyperpolarized Xe-129 resonances in living rats. Journal of Magnetic Resonance Series B 111: 300–304. 8661297 10.1006/jmrb.1996.0098

[30] Swanson SD, Rosen MS, Coulter KP, Welsh RC, Chupp TE (1999) Distribution and dynamics of laser-polarized Xe-129 magnetization in vivo. Magnetic Resonance in Medicine 42: 1137–1145. 10571936 10.1002/(sici)1522-2594(199912)42:6<1137::aid-mrm19>3.0.co;2-4

[31] Patz S, Muradian I, Hrovat MI, Ruset IC, Topulos G, et al (2008) Human pulmonary imaging and spectroscopy with hyperpolarized Xe-129 at 0.2T. Academic Radiology 15: 713–727. 18486008 10.1016/j.acra.2008.01.008 PMC2475597

[32] Driehuys B, Cofer GP, Pollaro J, Mackel JB, Hedlund LW, et al (2006) Imaging alveolar-capillary gas transfer using hyperpolarized Xe-129 MRI. Proceedings of the National Academy of Sciences of the United States of America 103: 18278–18283. 17101964 10.1073/pnas.0608458103 PMC1838742

[33] Lilburn DM, Pavlovskaya GE, Meersmann T (2013) Perspectives of hyperpolarized noble gas MRI beyond (3)He. J Magn Reson 229: 173–186. 23290627 10.1016/j.jmr.2012.11.014 PMC3611600

[34] Walker TG, Happer W (1997) Spin-exchange optical pumping of noble-gas nuclei. Review of Modern Physics 69: 629–642.

[35] Raftery D, Long H, Meersmann T, Grandinetti PJ, Reven L, et al (1991) High-Field NMR of Adsorbed Xenon Polarized by Laser Pumping. Physical Review Letters 66: 584–587. 10043847 10.1103/PhysRevLett.66.584

[36] Goodson BM (2002) Nuclear magnetic resonance of laser-polarized noble gases in molecules, materials, and organisms. Journal of Magnetic Resonance 155: 157–216. 12036331 10.1006/jmre.2001.2341

[37] Oros AM, Shah NJ (2004) Hyperpolarized xenon in NMR and MRI. Physics in Medicine and Biology 49: R105–R153. 15566166 10.1088/0031-9155/49/20/r01

[38] Cullen SC, Gross EG (1951) The Anesthetic Properties of Xenon in Animals and Human Beings, with Additional Observations on Krypton. Science 113: 580–582. 14834873 10.1126/science.113.2942.580

[39] Jameson CJ, Jameson AK, Hwang JK (1988) Nuclear-Spin Relaxation by Intermolecular Magnetic Dipole Coupling in the Gas-Phase - Xe-129 in Oxygen. Journal of Chemical Physics 89: 4074–4081.

[40] Driehuys B, Hedlund LW (2007) Imaging techniques for small animal models of pulmonary disease: MR microscopy. Toxicologic Pathology 35: 49–58. 17325972 10.1080/01926230601132048 PMC2747380

[41] Santyr GE, Lam WW, Parra-Robles JM, Taves TM, Ouriadov AV (2009) Hyperpolarized noble gas magnetic resonance imaging of the animal lung: Approaches and applications. Journal of Applied Physics 105..

[42] Faridy EE (1973) Effect of Hydration and Dehydration on Elastic Behavior of Excised Dogs Lungs. Journal of Applied Physiology 34: 597–605. 4574185 10.1152/jappl.1973.34.5.597

[43] Six JS, Hughes-Riley T, Stupic KF, Pavlovskaya GE, Meersmann T (2012) Pathway to Cryogen Free Production of Hyperpolarized Krypton-83 and Xenon-129. PLOS ONE 7: e49927. 23209620 10.1371/journal.pone.0049927 PMC3507956

[44] Hughes-Riley T, Six JS, Lilburn DML, Stupic KF, Pavlovskaya GE, et al.. (2013) Unpublished results.

[45] Cleveland ZI, Pavlovskaya GE, Elkins ND, Stupic KF, Repine JE, et al (2008) Hyperpolarized Kr-83 MRI of lungs. Journal of Magnetic Resonance 195: 232–237. 18948043 10.1016/j.jmr.2008.09.020

[46] Stupic KF, Elkins ND, Pavlovskaya GE, Repine JE, Meersmann T (2011) Effects of pulmonary inhalation on hyperpolarized krypton-83 magnetic resonance T-1 relaxation. Physics in Medicine and Biology 56: 3731–3748. 21628780 10.1088/0031-9155/56/13/001 PMC3249834

[47] Zhao L, Mulkern R, Tseng CH, Williamson D, Patz S, et al (1996) Gradient-echo imaging considerations for hyperpolarized Xe-129 MR. Journal of Magnetic Resonance Series B 113: 179–183. 11543610

[48] Kauczor HU, Markstaller K, Puderbach M, Lill J, Eberle B, et al (2001) Volumetry of ventilated airspaces by He-3 MRI preliminary results. Investigative Radiology 36: 110–114. 11224759 10.1097/00004424-200102000-00007

[49] Woodhouse N, Wild JM, van Beek EJR, Hoggard N, Barker N, et al (2009) Assessment of Hyperpolarized He-3 Lung MRI for Regional Evaluation of Interventional Therapy: A Pilot Study in Pediatric Cystic Fibrosis. Journal of Magnetic Resonance Imaging 30: 981–988. 19856418 10.1002/jmri.21949

[50] Martin C, Uhlig S, Ullrich V (1996) Videomicroscopy of methacholine-induced contraction of individual airways in precision-cut lung slices. European Respiratory Journal 9: 2479–2487. 8980957 10.1183/09031936.96.09122479

[51] Sturton RG, Trifilieff A, Nicholson AG, Barnes PJ (2008) Pharmacological characterization of indacaterol, a novel once daily inhaled beta(2) adrenoceptor agonist, on small airways in human and rat precision-cut lung slices. Journal of Pharmacology and Experimental Therapeutics 324: 270–275. 17916760 10.1124/jpet.107.129296

[52] Ressmeyer AR, Larsson AK, Vollmer E, Dahlen SE, Uhlig S, et al (2006) Characterisation of guinea pig precision-cut lung slices: comparison with human tissues. European Respiratory Journal 28: 603–611. 16737991 10.1183/09031936.06.00004206

[53] Patra AL (1986) Comparative anatomy of mammalian respiratory tracts: the nasopharyngeal region and the tracheobronchial region. J Toxicol Environ Health 17: 163–174. 10.1080/15287398609530813 3959107

[54] Lauzon A, Martin J (2008) Airway smooth muscle in experimental models. In: Chung KF, Airway Smooth Muscle in Asthma and COPD: Biology and Pharmacology: John Wiley & Sons 160–179.

[55] Lai YL, Hildebrandt J (1978) Respiratory mechanics in the anesthetized rat. J Appl Physiol 45: 255–260. 681212 10.1152/jappl.1978.45.2.255

[56] Takezawa J, Miller FJ, Oneil JJ (1978) Lung-Volumes and Single Breath Diffusing-Capacity for Carbon-Monoxide Measured in Small Laboratory Mammals. American Review of Respiratory Disease 117: 405–405.629475

[57] Kleinman LI, Siebens AA, Poulos DA (1964) Minimal Air in Dogs. Journal of Applied Physiology 19: 204–&. 10.1152/jappl.1964.19.2.204 14155282

[58] Horie T, Ardila R, Hildebra J (1974) Static and Dynamic Properties of Excised Cat Lung in Relation to Temperature. Journal of Applied Physiology 36: 317–322. 4814300 10.1152/jappl.1974.36.3.317

[59] Hughes JMB, Rosenzweig DY (1970) Factors Affecting Trapped Gas Volume in Perfused Dog Lungs. Journal of Applied Physiology 29: : 332–&. 10.1152/jappl.1970.29.3.332 4916039

[60] Frazer DG, Stengel PW, Weber KC (1979) Effect of Pulmonary-Edema on Gas Trapping in Excised Rat Lungs. Respiration Physiology 38: 325–333. 523848 10.1016/0034-5687(79)90058-6

[61] Milic-Emili J, Henderson J, Dolovich MB, Trop D, Kaneko K (1966) Regional Distribution of Inspired Gas in Lung. Journal of Applied Physiology 21: 749–759. 5912744 10.1152/jappl.1966.21.3.749

[62] Michels DB, Friedman PJ, West JB (1979) Radiographic Comparison of Human-Lung Shape during Normal Gravity and Weightlessness. Journal of Applied Physiology 47: 851–857. 511694 10.1152/jappl.1979.47.4.851

[63] Ball WC, Newsham LGS, Stewart PB, Bates DV (1962) Regional Pulmonary Function Studied with Xenon133. Journal of Clinical Investigation 41: : 519–&. 10.1172/JCI104505 PMC290945 13864417

[64] Lam WW, Holdsworth DW, Du LY, Drangova M, McCormack DG, et al (2007) Micro-CT imaging of rat lung ventilation using continuous image acquisition during xenon gas contrast enhancement. Journal of Applied Physiology 103: 1848–1856. 17690202 10.1152/japplphysiol.00009.2007

[65] Marcucci C, Nyhan D, Simon BA (2001) Distribution of pulmonary ventilation using Xe-enhanced computed tomography in prone and supine dogs. Journal of Applied Physiology 90: 421–430. 11160037 10.1152/jappl.2001.90.2.421

[66] Mansson S, Deninger AJ, Magnusson P, Pettersson G, Olsson LE, et al (2005) He-3 MRI-based assessment of posture-dependent regional ventilation gradients in rats. Journal of Applied Physiology 98: 2259–2267. 15640396 10.1152/japplphysiol.00245.2004

[67] Couch MJ, Ouriadov A, Santyr GE (2012) Regional ventilation mapping of the rat lung using hyperpolarized (129) Xe magnetic resonance imaging. Magn Reson Med. 10.1002/mrm.24152 22231781

[68] Kyriazis A, Rodriguez I, Nin N, Izquierdo-Garcia JL, Lorente JA, et al (2012) Dynamic Ventilation He-3 MRI for the Quantification of Disease in the Rat Lung. Ieee Transactions on Biomedical Engineering 59: 777–786. 22167560 10.1109/TBME.2011.2179299

[69] Eidelman DH, Dimaria GU, Bellofiore S, Wang NS, Guttmann RD, et al (1991) Strain-Related Differences in Airway Smooth-Muscle and Airway Responsiveness in the Rat. American Review of Respiratory Disease 144: 792–796. 1928950 10.1164/ajrccm/144.4.792

[70] Wang CG, Almirall JJ, Dolman CS, Dandurand RJ, Eidelman DH (1997) In vitro bronchial responsiveness in two highly inbred rat strains. Journal of Applied Physiology 82: 1445–1452. 9134891 10.1152/jappl.1997.82.5.1445

[71] Kirsch RA (1971) Computer Determination of Constituent Structure of Biological Images. Computers and Biomedical Research 4: : 315–&. 10.1016/0010-4809(71)90034-6 5562571

